# Progress in gallbladder cancer with lymph node metastasis

**DOI:** 10.3389/fonc.2022.966835

**Published:** 2022-08-22

**Authors:** Yuhang Li, Yinghui Song, Yujing Zhang, Sulai Liu

**Affiliations:** ^1^ Department of Hepatobiliary Surgery, The First Affiliated Hospital of Hunan Normal University, Changsha, China; ^2^ Central Laboratory of Hunan Provincial People's Hospital/The First Affiliated Hospital of Hunan Normal University, Changsha, China; ^3^ Key Laboratory of Molecular Epidemiology of Hunan Province, School of Medicine, Hunan Normal University, Changsha, China

**Keywords:** gallbladder cancer, lymph node metastasis (LNM), staging, treatment, surgery

## Abstract

Gallbladder cancer (GBC) is a malignant tumor that originates from the mucosal lining of the gallbladder. It is distinctly regional and is common in certain geographic regions of developing countries. GBC has a high degree of insidiousness as well as a high propensity for metastatic spread, resulting in the majority of patients being diagnosed at an advanced stage. Lymph node metastasis (LNM) is fairly common in GBC patients and is an independent risk factor for a poor prognosis. This article is focused on the lymph node pathways and metastatic directions of GBC. Furthermore, it summarizes the different lymph node groupings, disease stages and treatments. In the future, it is of great significance to develop individualized treatment and predict the outcomes of GBC patients with different lymph node conditions.

## Introduction

Primary gallbladder cancer (GBC) is one of the most common malignant tumors of the biliary system, and its incidence ranks fifth among the common malignant tumors of the digestive system (1, 2). GBC is a very rare disease in Western countries, but in some parts of the world, such as northeastern and central India, east Asia (Korea, Japan, China), central Europe (Slovakia, Poland, Czech Republic) and South America (Chile, Bolivia, Colombia), it has a high to moderate incidence (3, 4).

To date, various genetic variants, as well as different environmental factors, have been associated with a higher risk of developing GBC (**Table 1**). For example, chronic cholecystitis, gallstones, gallbladder polyps, obesity, various dietary factors, exposure to certain chemicals or metals, and infection with Salmonella are risk factors for GBC (6, 7). Additionally, women are 2-3 times more likely than men to develop GBC, possibly due to hormones that increase cholesterol levels in the bile, which in turn lead to the development of gallstones (8). The latest evidence shows (9) that the expression of the oestrogen/progesterone receptor is related to early tumorigenesis, indicating that oestrogen and progesterone may be involved in the carcinogenesis of GBC. GBC is occult in the early stages, lacks specific clinical manifestations, and has a low diagnosis rate (10–12). The overall prognosis is very poor (13–15), with a 5-year survival rate of 29.3% and a stage IV survival rate of only 1.3%. Once diagnosed and conditions permitting, radical surgery should be performed as soon as possible, and lymph node dissection should be performed if necessary (16).

**Table 1:** Patient predisposition, environmental factors and patient factors/conditions in GBC (5).

Patient predisposition	Environmental factors	Patient factors/conditions
Asia/South America	Geography	Diabetes
Age>65	Aflatoxins	Obesity
Female	Arsenic	Porcelain gallbladder
Genetics (variants)	Liver fluke	Primary sclerosing cholangitis
	Salmonella Infections	Gallbladder polyps
	Ochratoxin	Anomalous biliary ductal insertion
		Gallstones/Chronic cholecystitis
		Crohn’s disease
		Sjogren’s syndrome

Lymph node metastasis (LNM) is one of the most common types of GBC metastasis and is mainly related to the abundant lymphatic vessels in the subserosal layer of the gallbladder. It is also the most important factor affecting the clinical staging of GBC (17). It has been reported that radical (expanded or marginal dissection) cholecystectomy that removes the gallbladder along with normal liver tissue at the margins and regional lymphadenectomy with adequate lymph node dissection (LND) at the hepatic hilum to evaluate 6 or more regional lymph nodes can improve the survival rate (17–19). However, this surgery method removes many normal lymph nodes, making surgery more difficult and riskier. The metastatic status of LNM is an important factor in determining the surgical approach for GBC patients and affects the prognosis of the patients after radical resection (20, 21). Therefore, individualized treatment plans should be developed according to the specific conditions of different GBC patients. At present, there is no consensus on the scope of LND for GBC. Based on this, this article discusses different LNM pathways, staging, and LND protocols for GBC.

## GBC lymph node path and direction of metastasis

**Figure 1:**
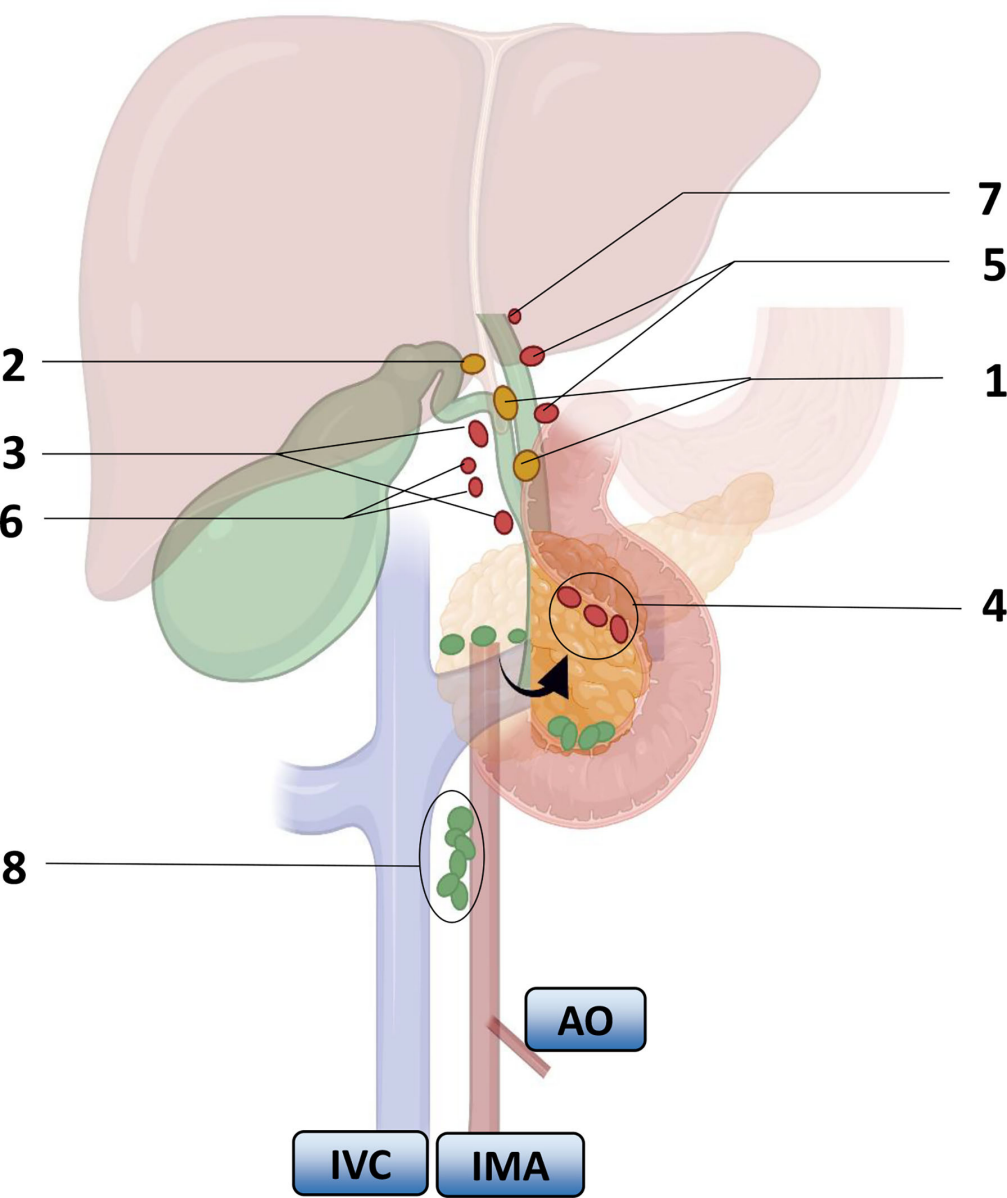
Lymph node substations and return routes for gallbladder cancer metastasis. Arrows indicate turning the pancreas head over. Numbers represent lymph nodes: 1. peri-biliary lymph nodes, 2. bile duct lymph nodes, 3. peri-portal lymph nodes, 4. lymph nodes behind the pancreaticoduodenum and above the head of the pancreas, 5. peri-hepatic artery lymph nodes, 6. peri-hepatic right artery lymph nodes, 7. hilar lymph nodes, 8. interaortocaval nodes. IVC: inferior vena cava, IMA: inferior mesenteric artery, AO: aorta. Color: First-order lymph nodes (yellow), second-order nodes (red), distal nodes (green).

Researchers carried out a series of studies on the distribution of lymph nodes near the gallbladder in the 1990s. Ito (22), Shirai (23) and Uesaka (24) gradually perfected the theory of gallbladder lymph node reflux, determined the extent of regional lymph nodes, and divided the lymph nodes of GBC into four stations (**Figure 1**). The guidelines for the diagnosis and treatment of GBC (2015 edition) pointed out that No. 13a was located at the demarcation points between the first and second lymph nodes of LNM of GBC, and No. 16 was located at the endpoint of LNM of GBC. Therefore, the order and scope of LND for GBC were based on the demarcation point of the lymph node substations. The Japanese Society for Gastric Cancer describes the information of each group of lymph nodes (**Table 2**), of which No. 12 and No. 8 are regional lymph nodes that have been agreed upon by the eighth edition of The American Joint Committee on Cancer (AJCC) and the guidelines of the Chinese Medical Association.

**Table 2:** Anatomical definition of lymph node stations (25).

No	Definition
1	Right paracardial LNs, including those along the 1st branch of the ascending limb of the left gastric artery
2	Left paracardial LNs including those along the esophagocardiac branch of the left subphrenic artery
3a	Lesser curvature LNs along the branches of the left gastric artery
3b	Lesser curvature LNs along the 2nd branch and distal part of the right gastric artery
4sa	Left greater curvature LNs along the short gastric arteries (perigastric area)
4sb	Left greater curvature LNs along the left gastroepiploic artery (perigastric area)
4d	Right greater curvature LNs along the 2nd branch and distal part of the right gastroepiploic artery
5	Suprapyloric LNs along the 1st branch and proximal part of the right gastric artery
6	Infrapyloric LNs along the 1st branch and proximal part of the right gastroepiploic artery down to the confluence of then right gastroepiploic vein and the anterior superior pancreatoduodenal vein
7	LNs along the trunk of left gastric artery between its root and the origin of its ascending branch
8a	Anterosuperior LNs along the common hepatic artery
8p	Posterior LNs along the common hepatic artery
9	Coeliac artery |
10	Splenic hilar LNs including those adjacent to the splenic artery distal to the pancreatic tail, and those on the roots of the short gastric arteries and those along the left gastroepiploic artery proximal to its 1st gastric branch
11p	Proximal splenic artery LNs from its origin to halfway between its origin and the pancreatic tail end |
11d	Distal splenic artery LNs from halfway between its origin and the pancreatic tail end to the end of the pancreatic tail
12a	Hepatoduodenal ligament LNs along the proper hepatic artery, in the caudal half between the confluence of the right and left hepatic ducts and the upper border of the pancreas
12b	Hepatoduodenal ligament LNs along the bile duct, in the caudal half between the confluence of the right and left hepatic ducts and the upper border of the pancreas
12p	Hepatoduodenal ligament LNs along the portal vein in the caudal half between the confluence of the right and left hepatic ducts and the upper border of the pancreas
13	LNs on the posterior surface of the pancreatic head cranial to the duodenal papilla
14	LNs along the superior mesenteric vein
15	LNs along the middle colic vessels
16a1	Paraaortic LNs in the diaphragmatic aortic hiatus
16a2	Paraaortic LNs between the upper margin of the origin of the celiac artery and the lower border of the left renal vein
16b1	Paraaortic LNs between the lower border of the left renal vein and the upper border of the origin of the inferior mesenteric artery
16b2	Paraaortic LNs between the upper border of the origin of the inferior mesenteric artery and the aortic bifurcation

LNs, Lymph nodes.

The LNM of GBC depends on the direction of lymphatic drainage. Uesaka (24) et al. found that lymphatic drainage mainly passed through three pathways by injecting carbon particle suspension into the gallbladder wall during surgery. On the right route, it could be seen in 95% of the LNM patients; it passed through the lymph nodes around the common bile duct, to No. 13a or the lymph nodes around the portal vein, and returned to the lymph nodes around the aorta. On the left route, it could be seen in 50% of the LNM patients; the lymph returned to the gallbladder lymph nodes in the gallbladder triangle, then passed through the lymph nodes behind the head of the pancreas, passed through the lymph nodes of the hepatoduodenal ligament, and returned to the lymph nodes around the aorta. 3) The portal route was seen in 20% of the LNM patients, which drained directly through the hilar lymph nodes to the peri-aortic lymph nodes (23). They all converge in the para-aortic lymph nodes, among which the lymph nodes in the interaortic space can play an important role.

In addition, metastasis to the liver is also very common. If the tumor site of the GBC patients is adjacent to the gallbladder bed, LNM and direct invasion can both allow for spread to the liver. Accordingly, the regional lymph nodes of GBC can be defined as the groups of lymph nodes that return to the front of the periaortic lymph nodes (26). GBC also spreads mainly through the right side, while the left side pathway complements the lymphatic spread, and the right-side pathway meets the left side pathway behind the head of the pancreas, which also means that the involvement of lymph nodes around the head of the pancreas is a significant factor affecting the patient’s prognosis.

The Union for International Cancer Control (UICC) study pointed out that after referring to the lymphatic drainage pathways of the gallbladder, it was clear that there were 2 metastatic endpoints for lymph nodes, with the first endpoint (N1) being the common bile duct lymph nodes and gallbladder neck lymph nodes and the second endpoint (N2) being the remaining lymph nodes. However, the Japanese Society of Hepato-Biliary-Pancreatic Surgery (JSHBPS) had a different view on the classification of metastatic endpoints of lymph nodes. It agrees with the first metastatic endpoint of lymph nodes (N1) as indicated by the UICC but differs in the classification of the second metastatic endpoint of lymph nodes (N2), which considers posterior portal lymph nodes, common hepatic artery lymph nodes, and posterior supra-pancreaticoduodenal lymph nodes as the second metastatic endpoint of the lymph nodes. In addition, it proposed that there were more than 2 metastatic endpoints of lymph nodes, and there was a third metastatic endpoint (N3), including celiac artery lymph nodes, superior mesenteric artery lymph nodes and para-aortic lymph nodes. Therefore, we can conclude that GBC spreads most often from the gallbladder to the periportal lymph nodes and then to the abdominal aortic station but may also spread through the abdominal nodal station and then to more distant axial sites.

Considering the significant differences in the classification of LNM between the UICC and the JSHBPS, Chijiiwa (27) conducted an in-depth study. The survival of patients with N1 was roughly similar according to the classification of LNM by the Anti-Cancer Alliance and JSHBPS, while the survival of patients with N1 classified by JSHBPS was better than that of patients with N1 classified by the International Anti-Cancer Federation. The survival rate of patients with N3 was poor, which also indicated that the JSHBPS classification of LNM endpoints was more detailed. Kishi (28) also agrees with JSHBPS’s classification of LNM endpoints. He believes that such a classification method is more scientific and accurate, is more helpful for the formulation of patient treatment plans, and can comprehensively evaluate the prognosis of patients based on such a detailed classification. However, Europe and the United States do not accept such a classification and it is rarely used. In the future, we can refer more to the JSHBPS classification in our clinical work and compare it with the International Union Against Cancer’s classification in terms of the patient’s specific LNMs. This will lead to a more accurate and safe classification, which will effectively improve the subsequent staging, treatment and prognosis.

## Lymph node diagnosis of GBC

GBC lymph node radiomics can be correlated with tumor genetic features and protein phenotypes to predict the biological behavior of the tumor (29). Ultrasound, enhanced CT, MRI and 18-FDG PET-CT (30) are used to determine the LN status, and an LN diameter greater than 1 cm is considered a positive criterion for LNM in these examinations. However, some studies have reported that inflammation and metabolites in the area of the tumors in the biliary system or biliary obstruction may also lead to LN hyperplasia. Ultrasound can be used as a screening method for the diagnosis of GBC with an accuracy rate of 80% (31). CT has a remarkable effect in identifying the T-stage of GBC (32). The sensitivity of CT in distinguishing T1 from ≥T2, ≤T2 from ≥T3 and ≤T3 from T4 stage GBC was 79.3%, 92.7% and 100%, respectively, and the specificity was 98.8%, 86% and 100%, respectively (33). A meta-analysis reported that it is challenging to detect LNM smaller than 1 cm on MRI (34), although it is effective in predicting LNM in GBC. Currently, fine-needle aspiration biopsy remains the gold standard for preoperative diagnosis, but its applicability is limited. This approach is also associated with serious complications, such as bleeding, tumor spread, and lymphatic fistulae.

Currently, the presence of LNM in GBC is often difficult to determine preoperatively (35, 36). Petrowsky (37) found that the accuracy of enhanced CT and PET/CT for predicting regional LNM was 24% and 12%, respectively. In patients with confirmed or suspected GBC, 18-FDG PET-CT had a 56% sensitivity to detect occult peritoneal or omental LNM (38). The detection of occult LNM may help identify patients who will benefit from radical resection (39).

The radiomics model had the highest AUC values for accurately predicting preoperative gallbladder cancer LNM (40). In addition, surgeons can use nomograms to assess the need for LN resection before surgery, benefiting patients with actual LNM negativity (40).

The clinical application of indocyanine green (ICG) fluorescence imaging technology in biliary surgery has gradually highlighted its role. Currently, ICG fluorescence imaging is a promising tool for sentinel lymph node (SLN) detection in patients with breast, gastric and colorectal cancers (41, 42). In a recent study, it was feasible to attempt to detect SLN in GBC using a laparoscopic magnetic probe using the ICG and superparamagnetic iron oxide (SPIO) dual tracer approach (43). This study provides a potential bridge to the clinical application of our laparoscopic approach for the detection of SLN metastases in cancers of internal organs that are difficult to detect from the surface of the body or through endoscopy. There are advantages and disadvantages to each of the ICG intravenous or bile duct injection imaging modalities, and the choice of the optimal timing of administration and dose selection remains controversial and will continue to be explored in the future to fully exploit the value of ICG fluorescence imaging in biliary surgery, particularly in the imaging of LNM from GBC.

## Staging and lymph node dissection for GBC

Although the AJCC TNM staging system is the most commonly used lymph node staging system for GBC, this staging system has not been accepted worldwide due to controversies regarding the attribution of some lymph nodes (peripancreatic lymph nodes, abdominal trunk lymph nodes) and the division of lymph node substations in the JSBS staging system and TNM staging system (**Table 3**). Studies have shown that recurrence, extrahepatic metastasis and LNM are closely related to T stage, and among the metastases of GBC, LNM has the highest risk of recurrence. Studies have confirmed that the higher the T-stage of GBC is, the higher the probability of LNM, with a rate of T1a stage of 0-2.5% (44), T1b stage of 5%-16%, T2 stage of 9%~~30%, T3 stage of 39%-72%, and T4 stage rate as high as 67%-80% (45), and patients with positive LNM are directly classified as stage IIIb (46).

**Table 3:** Statements in different guidelines on the extent of lymph node dissection in gallbladder cancer.

Guidelines	Gallbladder cancer regional lymph nodes	Lymph node dissection range
China Guide	Include the common hepatic artery (No.8), the proper hepatic artery (No.12a), the common bile duct (No.12b),the gallbladder neck (No.12c), the hepatic hilum (No.12h), and the portal vein Posterior (No.12p), Posterior and superior of pancreatic head (No.13a)	No.8, 12a, 12b, 12c, 12h, 12p, 13a
Japanese JSBS Guide	Include hepatoduodenal ligament (No.12), next to common hepatic artery (No.8), posterior and superior of pancreatic head (No.13a)	No.8, 12, 13a
US NCCN Guide	Include cystic duct, common bile duct, hepatic artery, and paraportal lymph nodes. One to three positive lymph nodes are N1, and more than four are N2	The scope of dissection includes all lymph nodes in the hepatic portal area, and the number of lymph nodes dissected is 6

China: Guidelines for the Diagnosis and Treatment of Gallbladder Cancer from the Chinese Journal of Surgery; Japan: From the Japanese Society of Hepato-Biliary-Pancreatic Surgery; US: From the 2018 National; Comprehensive Cancer Network Clinical Practice Guidelines for Hepatobiliary Tumours.

Stage T1b is always a stage that needs attention. Although T1b is generally considered an early stage, patients in stage T1b are prone to develop local LNM. Therefore, LND is required for patients with stage T1b disease who have local LNM. Pawlik (47) found LNM in 12.5% of T1 cases in a series of complementary radical GBC resections; Vo (48) found LNM in 15% of T1b patients undergoing radical surgery using US cancer registry data; and Jensen (49) found LNM in 24% of T1b patients undergoing radical surgery. It can be inferred that the probability of developing LNM in T1b patients is approximately 20%. According to the AJCC gallbladder cancer staging system (5), the lymph nodes of the bile duct, common bile duct, portal vein and hepatic artery are regarded as the regional lymph nodes of GBC, and approximately 54.7% of patients have LNM, among which N1 metastasis and N2 metastasis are the most common (50). The N stage will be divided according to the number of metastatic lymph nodes, 1 to 3 LNM localization as N1 stage, and ≥ 4 positive lymph nodes defined as N2 stage. It is recommended that lymph node dissection include all lymph nodes in the hepatoportal and hepatoduodenal ligament areas, and the number of lymph nodes dissected should be over 6, which can help to accurately determine the N stage. This well reflects the extremely deplorable prognosis of GBC once LNM occurs. Recently, some researchers have proposed indicators for the assessment of LNM status, including lymph node ratio (LNR), positive lymph node count (PLNC), and positive lymph node location, where LNR is PLNC divided by the total number of lymph nodes removed. In contrast to the traditional N-staging of AJCC based on the location of lymph nodes, these three indicators consider the number of metastatic lymph nodes, which also provides a basis for future surgical requirements and accurate staging (51, 52).

Due to the different treatment modalities of LNM, a comprehensive evaluation is needed to develop a more appropriate treatment plan for patients (53). The NCCN has stated that LNM can inform the surgical approach and extent of resection for GBC (54). According to SEER data, only 5.3% of 2835 patients with T1-T3, M0 stage GBC who were surgically resected were able to have more than 3 LNs removed during surgery. Due to the complex anatomy surrounding the lymph node drainage pathway in GBC, incomplete dissection of lymph nodes compromises the outcome of the procedure (55). Data from high-volume centers showed that up to 26% of patients with GBC have axial LN involvement (main vena cava/abdominal cavity), which would negate any benefit of radical surgery, suggesting that trunk-inferior vena cava LN sampling should be routinely performed at the start of surgery (56).

**Figure 2:**
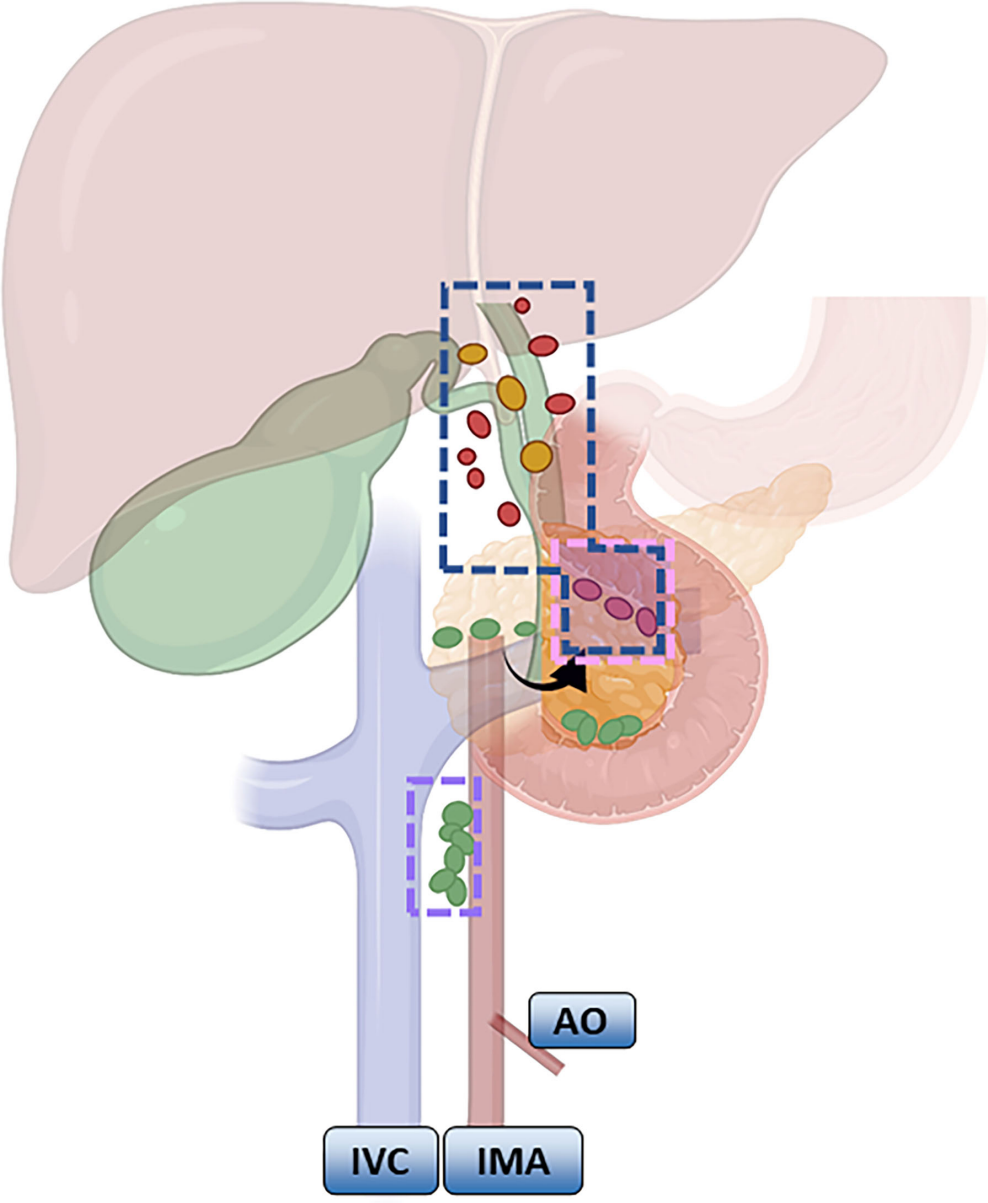
Meaning of lymph node regions: Blue: Routine lymph node dissection range; pink: the dividing point between the lymph nodes of the first station and the second station; purple: the focus of the lymph node station.

According to the NCCN guidelines, LND should be routinely performed for GBC that is above T1b. LND of the hepatoduodenal ligament is common in Europe and the United States (57, 58). The scope recommended by the Chinese Medical Association guidelines is generally consistent, and No. 13a and No. 16 lymph node biopsies are routinely recommended based on LND of the hepatoduodenal ligament. If No. 16 is positive, it indicates distal metastasis and is a contraindication to radical surgery; if No. 13a is positive or T3 stage and above, expanded LND, including No. 12, No. 8, No. 9 and No. 13, is recommended. Expanded LND is mostly seen in Japan, involving removal of the first and second station LNs (**Figure 2**). Shirai (59) pointed out that the posterior superior border of the pancreatic head needs to be clearly exposed after LND.

The scope of surgical resection in stage T2 includes the gallbladder and segment IVa+V of the liver, and the LND range is consistent with that in stage T1b. Stages T3 and T4 GBC are often accompanied by distant metastases due to their aggressiveness, and only a small proportion of patients have the opportunity to undergo surgery (60). In patients with resectable stage T3 or T4, extended LND removal is often needed. The posterior superior pancreatic head and para-aortic lymph nodes are important staging points. Among them, No. 13a lymph nodes are an important cause of LNM in GBC (61). Approximately 7.7%-33.1% of lymph node-positive GBC patients have No. 13a LNM. Although No. 13a lymph nodes are defined as distant metastases by the UICC and AJCC guidelines, there is no definitive clinical research evidence to confirm the definition (62, 63). At the same time, a series of studies by Japanese scholars recently suggested that the prognosis of No. 13a LNM is similar to that of regional LNM (64–66). However, previous studies have found that surgical treatment of patients with lymph node-positive GBC No. 13a is not effective, and its prognosis is similar to that of distant metastasis. If 13a positivity is regarded as distant metastasis, clinical trials have confirmed that gemcitabine combined with cisplatin chemotherapy can improve the prognosis of the patients (67). The treatment of these patients still needs to be further explored.

## Neoadjuvant and adjuvant therapy for GBC

Neoadjuvant and adjuvant therapies are gradually entering the treatment options for GBC patients and are expected to be a new treatment strategy to improve the prognosis of GBC. Neoadjuvant therapy strategies have led to surgical access for some patients with unresectable, locally progressive GBC (68). An ongoing phase III clinical trial (GAIN trial) aims to validate the advantage of neoadjuvant therapy in patients with histopathologically confirmed GBC after cholecystectomy. The regimen is 3 cycles of preoperative and postoperative GC regimen (cisplatin in combination with gemcitabine) combined with surgery, with the primary results to be published in 2024 (69). Another ongoing phase III RCT (POLCAGB study) compared the efficacy of neoadjuvant chemoradiotherapy with neoadjuvant chemotherapy alone to verify whether neoadjuvant radiotherapy has advantages in reducing the tumor stage and improving OS (70).

In addition, an increasing number of researchers are paying attention to the efficacy of adjuvant therapy after surgery in GBC. In 2012, Horgan analyzed the efficacy of adjuvant chemotherapy and radiotherapy in patients after biliary tract cancer, including GBC resection, and their findings showed that adjuvant therapy might be beneficial in patients with biliary tract cancer with positive margins (R1) and lymph node invasion (stage N1) (71). In a study conducted from January 2012 to December 2014, a total of 40 patients with GBC were evaluated before treatment with CT scans, showing LN involvement (n=34) and no lymph node enlargement (n=6). After neoadjuvant therapy, 66.6% and 83% of the liver and lymph node staging declined, respectively, and the patients underwent extended cholecystectomy successfully. The histopathological CR rates for the liver and lymph nodes were 16.6% and 83.3%, respectively. All resections reached R0. Neoadjuvant therapy for unresectable GBC resulted in a 15% resectability rate. This approach has great potential for achieving R0 and lymph node-negative disease. The radiological step-down for lymph node involvement was 67.5%.

Lymph node regression can be used as a predictor of the response to neoadjuvant therapy (72). Wu et al. showed that camrelizumab + AG could be a new treatment option for GBC with multiple abdominal LNM (73, 74). The ongoing European phase III clinical trial (ACTICCA-1 study, NCT02170090) is exploring the efficacy of adjuvant therapy with the GC regimen. A total of 781 patients with postsurgical resection biliary tract cancer were enrolled in the program, and disease-free survival (DFS) was the primary study endpoint (75). Ozer performed a cohort study in which out of 6391 patients who underwent radical surgery for GBC. Patients with localized or locoregionally advanced gallbladder cancers were categorized as receiving neoadjuvant chemotherapy, adjuvant chemotherapy, or surgery alone. Adjuvant or neoadjuvant chemotherapy was used more frequently than surgery in patients with lymph node-positive cancers [1482 (67.2%) vs. 53 (65.4%) vs. 912 (49.7%)]. Among patients with lymph node-positive GBC, neoadjuvant therapy combined with surgery was associated with longer median overall survival than patients who underwent surgery alone (30 months vs. 14 months, P = 0.002). In this cohort study, the use of adjuvant and neoadjuvant chemotherapy was low in patients with surgically resected GBC. Chemotherapy was used more frequently than surgery in lymph node-positive disease than in lymph node-negative disease. Adjuvant and neoadjuvant chemotherapy were associated with increased survival for lymph node-positive GBC. These findings suggest that adjuvant chemotherapy and neoadjuvant chemotherapies should be considered in the treatment of GBC, especially when there is LNM (76).

Today, most surgeons have greater expectations of neoadjuvant therapy. Its advantages include reducing the primary tumor to improve the R0 resection rate and having a therapeutic effect on small distant metastases that are difficult to detect on imaging. However, the timing and duration of neoadjuvant therapy, as well as the safety of extensive surgery, such as extended hepatectomy and/or pancreaticoduodenectomy, should also be more fully explored and evaluated in future clinical trials. In addition, there are still some problems with adjuvant therapy after biliary tract cancer resection. The BILCAP study showed a survival advantage for patients treated with postoperative adjuvant therapy but did not assess the issue of impaired liver function or reduced physical status scores due to extensive hepatectomy, resulting in worse tolerance of adjuvant therapy. This group of patients may not benefit from postoperative adjuvant therapy, and many studies have failed to mention the proportion and number of patients in this group. Additional studies are expected to confirm these results.

## Prognosis of GBC treatment

The insidious onset of GBC makes the early diagnosis of GBC difficult. LNM is an independent predictive factor for GBC patient prognosis (77, 78). LND is recommended after surgical resection of ≥T1b GBC, and its therapeutic value is well confirmed. For advanced GBC patients with LNM or palliative surgery only, the prognosis is poor.

TNM staging is correlated with the prognosis of patients. With the increase of the T stage, the possibility of the patient developing LNM becomes greater. The rates of LNM in T1a and T1b are 0-2.5% and 5%-16%, respectively, 9%-30% in T2, and over 62.7% in T3 and T4 (45, 79, 80). The higher the stage, the worse the prognosis of the patient and the decreasing 5-year survival rate (81–83). The 5-year survival rate of T1a patients ranged from 44.7% to 100%, and the effect of LNM on the 5-year survival rate of patients with stage T1a was inconclusive (44).

Patients with stage T2 disease have a higher risk of LNM and are not well served by cholecystectomy alone. In the Chio study (84), a comprehensive analysis of 83 patients with stage T2 disease found that vascular invasion, good tumor differentiation, LND, and R0 eradication were all independent predictive factors for the prognosis of patients with stage T2 disease. It has been shown that approximately 30% of patients with stage T2 have LNM, and only 40% of patients with stage T2 can survive for more than 5 years if they are treated with simple cholecystectomy, while more than 80% of patients with stage T2 can survive for more than 5 years if they are treated with radical cholecystectomy (85, 86).

It has also been shown that the overall survival rate of patients with stage T1b-T3 GBC who underwent LND was significantly improved compared to radical surgery without LND, and there was no significant difference in the survival rate between patients who did not undergo LND and those who underwent cholecystectomy alone (49). Birnbaum determined (87) the value of LNR in assessing patient outcomes in a study of 112 cases, with cholecystectomy alone being significantly less effective and radical surgery being the best approach. The results also showed that the earlier the lymph node stage was, the better the prognosis.

Patients with N1 lymph node involvement have been reported to survive for a long time. In contrast, those with N2 lymph node involvement have no significant survival benefit (56). LND includes N1 (No.12a, No.12b, No.12c and No.12p) and N2 (No.8 and No.13a) station lymph nodes. N1 positivity leads to a significant decrease in the efficacy of surgical resection; N2 positivity marks the inability to perform radical surgical resection, leading to increased complications, morbidity and mortality. Regional lymph node positivity is a predictor of a worse prognosis in GBC. Radical cholecystectomy, including extended systemic LND, is recommended to improve the outcomes of the procedure (46). Nevertheless, not all patients will benefit from radical LND. Previous studies have reported that patients with negative LNM should not undergo extended radical resection, as this may lead to serious postoperative complications (56).

For the number of lymph nodes cleared, both the Ito (18) and NEGI (88) studies found that at least six lymph nodes needed to be cleared and examined in patients with negative LNM to obtain a better postoperative staging of patients. The American Hepato-Pancreato-Biliary Expert Consensus also concluded that lymph node positivity was an important predictor of postoperative survival in GBC and that adequate and accurate staging required the removal of at least six lymph nodes (19). These studies all suggested that clearing a sufficient number of lymph nodes not only contributed to more accurate staging of LNM status in GBC but also improved the patient prognosis without increasing the postoperative complications. Patients with at least 6 lymph nodes removed with N0 stage had significantly improved 5-year disease-specific survival compared with N0 stage patients based on fewer nodes (72% vs. 45% p<0.01) (89). Patients with N2 LNM who underwent radical rescetion had a poor prognosis, and palliative treatment maybe the better option (88). Wu believes that adequate lymph node dissection should be performed, with at least 15 lymph nodes detected, to ensure an accurate assessment of patient prognosis (90). However, in actual clinical practice, it is difficult to guarantee that all patients can have a sufficient number of lymph nodes dissected due to differences in individual patients and surgical approaches, which definitely affects the accurate determination of the number of LNMs and thus the accuracy of N staging. According to the Chinese Society of Biliary Surgery, extended lymph node dissection is indicated for periabdominal trunk lymph nodes, peri-pancreatic head lymph nodes, common hepatic artery lymph nodes, and duodenal ligament lymph nodes in patients with N1 LNM from GBC.

For patients with T1b, the value of lymph node dissection still needs to be explored (91). In a nationwide retrospective cohort of GBC in Korea, Lee et al. found that in patients with T1b GBC, radical surgery for GBC did not provide a significantly better prognosis than cholecystectomy alone (92), and the Korean Hepatobiliary and Pancreatic Society also had reservations about radical surgery for T1b in their guidelines (93). However, most scholars still advocate radical surgery for GBC at stage T1b. The main argument is that because of the invasion of the muscular layer at this stage, there is no peritoneal coverage between the gallbladder bed and the liver, so the tumor cells can easily invade the liver. In a single-center retrospective study, Pawlik found residual tumors in 37.5% of T1 patients who underwent supplemental radical surgery after cholecystectomy (47). Additionally, database information in the US showed that radical surgical resection in T1b patients was beneficial (94). The 5-year survival rates vary widely among studies, ranging from 50% to 100% (48, 95–97). The 5-year survival rate of 70.5% for patients with T1b GBC in the Shanghai Xinhua Hospital data study was similar to that reported by Vo based on the US tumor Registry (48) but significantly lower than the 89.0% reported by Kim (96) and the 90.4% reported by Yoon (95). This survival difference may be due to the different proportions of lymph node-positive patients between the cohorts. Vo reported a higher rate of lymph node positivity of 15% (48), whereas the Kim (96) and Yoon (95) cohorts had significantly lower rates of lymph node positivity (5.8% and 0%). In a Japanese gallbladder cancer cohort that included 172 hospitals, the rate of positive lymph nodes in T1b patients was 15.6%, and their 5-year survival rate was 72.5% (97). This finding also demonstrates that as the rate of positive lymph nodes increases, the five-year survival rate of patients decreases accordingly. Therefore, radical surgery should be performed in patients with T1b, and evidence to support the guidelines needs to be improved in future clinical work.

## Conclusion

Late diagnosis and a poor prognosis are major problems for GBC treatment. LNM and debulking of GBC have become the focus of research. In the process of gallbladder LNM, the lymph nodes along the common bile duct and gallbladder triangle are the first to be involved, so it is necessary to completely remove the lymph nodes in this area when performing radical treatments on patients.

The higher the histologic grade and T-stage are, the more likely LNM will occur. In the surgical treatment of patients with LNM from GBC, we need to clarify most of the relevant indicators, including the number of lymph nodes, positive rate and extent, and we need to distinguish the adjacent tissues clearly to make the lymph node dissection more accurate and effective to achieve R0 clearance of the tumor. We also have to improve the accuracy of the lymphatic tracing technique to achieve the purpose of staining the lymph nodes *in vivo*, assisting in complete lymph node dissection, effectively protecting the surrounding adjacent tissues, improving the efficiency of surgery, reducing the risk of recurrence, and prolonging the patient’s survival time.

Currently, there are limitations in the diagnosis and treatment of gallbladder cancer with LNM. In terms of diagnosis, ultrasound, CT, MRI and other techniques are not accurate enough to detect early lymph node invasion, and there is a high rate of misdiagnosis for lymph node enlargement of unknown origin. Lymph node biopsy is the gold standard for lymph node diagnosis, which reduces the false-positive rate but increases the risk of lymph node fistula. The use of ICG can improve the detection of positive lymph nodes intraoperatively, but a large number of clinical trials are still needed to find a safer and more efficient dose and modality. In terms of treatment, the detailed scope of intraoperative lymph node dissection remains an area of concern for further research and standardization. Moreover, the development of more standardized and efficient neoadjuvant and postoperative adjuvant therapy protocols still requires a great deal of trial and error.

In the future, with further research on the LNM pathways and the analysis and comparison of more clinical samples, it is possible to combine appropriate neoadjuvant and adjuvant therapy according to the specific conditions of LNM in patients with GBC, which is of great significance for the diagnosis and treatment of GBC.

## Author contributions

YL and YS wrote the manuscript. YL and YZ made pictures and tables. SL helped perform the analysis with constructive discussions. All authors contributed to the article and approved the submitted version.

## Funding

This work was financially supported by the following funds: Youth Talent of Hunan Province (2020RC3066), Hunan Natural Science Fund for Excellent Young Scholars (2021JJ20003), Hunan Provincial Natural Science Foundation of China (2020JJ5610), Hunan Provincial Development and Reform Commission Project (2019FGW26), Education fund item of Hunan Province (20B380), Project of Hunan Provincial Health Commission (202104010997), Chen Xiao-Ping Foundation for the Development of Science and Technology of Hubei Province (CXPJJH12000001-2020322).

## Conflict of interest

The authors declare that the research was conducted in the absence of any commercial or financial relationships that could be construed as a potential conflict of interest.

## Publisher’s note

All claims expressed in this article are solely those of the authors and do not necessarily represent those of their affiliated organizations, or those of the publisher, the editors and the reviewers. Any product that may be evaluated in this article, or claim that may be made by its manufacturer, is not guaranteed or endorsed by the publisher.
